# The Effect of Aerobic Exercise Training on Patients with Type III Spinal Muscular Atrophy

**DOI:** 10.3390/jcm14176087

**Published:** 2025-08-28

**Authors:** Sezan Mergen Kilic, Fatma Mutluay, Arman Cakar, Murat Giris, Hacer Durmus, Ilknur Bingul, Asuman Gedikbasi, Canan Kucukgergin, Zehra Oya Uyguner, Yesim Parman

**Affiliations:** 1 Neuromuscular Unit, Istanbul Faculty of Medicine, Istanbul University, 34093 Istanbul, Turkey; arman.cakar@istanbul.edu.tr (A.C.); hacer.durmus@istanbul.edu.tr (H.D.); parmany@istanbul.edu.tr (Y.P.); 2 Department of Physiotherapy and Rehabilitation, Institute of Health Sciences, Istanbul Medipol University, 34214 Istanbul, Turkey; 3 Department of Physiotherapy and Rehabilitation, Faculty of Health Sciences, Yalova University, 77200 Yalova, Turkey; fatma.mutluay@yalova.edu.tr; 4 Department of Medical Biochemistry, Istanbul Faculty of Medicine, Istanbul University, 34093 Istanbul, Turkey; mgiris@istanbul.edu.tr (M.G.); ilknur.bingul@istanbul.edu.tr (I.B.); ckgergin@istanbul.edu.tr (C.K.); 5 Department of Pediatric Basic Sciences, Institute of Child Health, Istanbul University, 34093 Istanbul, Turkey; asuman.gedikbasi@istanbul.edu.tr; 6 Department of Medical Genetics, Istanbul Faculty of Medicine, Istanbul University, 34093 Istanbul, Turkey; o.uyguner@istanbul.edu.tr

**Keywords:** SMA Type III, physiotherapy, aerobic exercise, SMN protein, IGF-1, functional performance, exercise capacity, neuromuscular disorders

## Abstract

**Background:** Spinal muscular atrophy (SMA) is a neurodegenerative disorder caused by variants in the *SMN1* gene. This study investigates the functional and biochemical effects of moderate-intensity aerobic exercise in SMA Type III patients. **Methods:** Twenty-three patients aged 18–57 years were included in this study. The training group underwent a 12-week aerobic exercise program using a bicycle ergometer at 60–70% of their maximum heart rate three times per week for 30 min per session. The training continued for an additional four months. The primary outcome measures were the six-minute walk distance and oxygen uptake, both reflecting exercise capacity. Secondary outcome measures included muscle strength with dynamometer, functional performance, and fatigue with different scales. Furthermore, serum survival motor neuron (SMN) protein and insulin-like growth factor-1 (IGF-1) hormone levels were measured at baseline, post-training first measurement (after 12 weeks), and post-training second measurement (after 28 weeks). **Results:** The exercise group showed a significant increase in exercise capacity (*p* < 0.001) and 6MWT walking distance (*p* = 0.003). Furthermore, reduction in walking time in the 10-m walk test (*p* = 0.019) and improvements in strength of the right and left quadriceps (*p* = 0.004, *p* = 0.031) and right gastrocnemius (*p* = 0.034) muscles were identified. Furthermore, an improvement in the Fatigue Severity Scale (FSS) (*p* = 0.037) was found. SMN protein and IGF-1 levels were increased in the second measurement in the training group (*p* = 0.022 and *p* = 0.016, respectively). **Conclusions:** An aerobic exercise program improved physical function and muscle strength and reduced fatigue in SMA Type III patients, with sustained biochemical improvements. Aerobic exercise may serve as a beneficial adjunct therapy for this population.

## 1. Introduction

Spinal muscular atrophy (SMA) is an autosomal recessive hereditary disease characterized by the degeneration of alpha motor neurons. This leads to progressive muscle atrophy and weakness, affecting approximately 1 in 6000 to 10,000 infants [1,2]. SMA occurs due to homozygous mutations in the *SMN1* gene located in the 5q13 region. This results in insufficient survival motor neuron (SMN) protein expression [3]. The SMN protein plays a crucial role in maintaining motor neurons [4]. It is primarily produced by the *SMN1* gene, with only 10–15% being generated by the *SMN2* gene. The *SMN2* gene differs from *SMN1* by a single nucleotide in exon 7, which leads to the exclusion of exon 7 during splicing and the production of a nonfunctional SMN protein [5]. The disease is classified into four phenotypes, ranging from the most severe (Type I) to the mildest (Type IV), based on the age of onset and motor function. Children with Type III SMA can walk independently, but their motor functions vary [6].

Therapeutic strategies aim to increase SMN protein levels to reduce the severity of the disease [7,8]. Nusinersen, the first approved treatment for SMA, is an intrathecally administered antisense oligonucleotide (ASO) designed to bind to the pre-mRNA of *SMN2* and promote the inclusion of exon 7 [9]. Following the introduction of nusinersen, there has been an increased focus on physical therapy, emphasizing its importance for individuals diagnosed with SMA [10]. Regular exercise is one of the recommended approaches for the standard care of SMA patients [11]. Historically, individuals with SMA and other motor neuron disorders, such as amyotrophic lateral sclerosis (ALS), were discouraged from doing regular exercise. However, studies on SMA mouse models have demonstrated that running-based exercise increases the amount of SMN transcripts containing exon 7 in the spinal cords of trained mice. These studies reported exercise-specific benefits, including increased motor neuron survival, extended lifespan, and preserved muscle strength. Preliminary evidence from SMA animal models provides the first indication of exercise’s beneficial effects on SMA [12,13,14]. In a recent study in children with SMA Types II and III, progressive strengthening exercises showed trends of improvement in strength and motor function, demonstrating that such interventions are feasible, safe, and well-tolerated [15]. While supportive evidence for aerobic exercise in SMA is limited, studies have shown the safety of aerobic exercise in clinically diverse SMA populations [16,17,18,19,20]. Recent studies indicate that a 12-week cycling ergometer training program in SMA Type III patients did not cause muscle damage but increased maximal oxygen uptake (VO2 max). However, fatigue-related challenges were noted among patients [17]. Exercise influences growth hormones such as insulin-like growth factor 1 (IGF-1), which has neuroprotective effects on motor neurons. IGF-1 is a critical factor for motor neuron survival and maintenance [14,21,22]. The impact of exercise on IGF-1 in SMA patients has not yet been demonstrated. In one study, while improvements in physical function were observed in SMA Type II patients after arm cycling exercises, no exercise-induced changes were found in SMN protein, IGF-1, or insulin-like growth factor-binding protein 3 (IGFBP3) levels [18].

The aim of this study was to observe the effect of moderate-intensity aerobic exercise training on physical function in ambulatory SMA Type III patients and to investigate the molecular response of SMN protein IGF-1 hormone to exercise.

## 2. Materials and Methods

### 2.1. Participants and Ethical Approval

All procedures were conducted in accordance with the principles of the Declaration of Helsinki. The study was conducted at the Neuromuscular Unit of Neurology Department of Istanbul Medical Faculty. It was approved by the university’s “Clinical Research Ethics Committee” (28 January 2022-728866).

Out of a total of 23 patients aged 18–57 years with genetically confirmed Type III SMA receiving maintenance treatment with nusinersen, 12 patients were assigned to the training group, and 11 were assigned to the home-exercise group. Patients who were able to walk at least 25 m were included. Patients with severe medical and/or physical limitations that could prevent participation in exercise were excluded. Participants were not randomly assigned. Instead, to ensure baseline comparability between the groups, patients in the training and home-exercise groups were pair-matched based on age, sex, and baseline HFMSE score. Baseline similarity between groups was assessed using descriptive statistics, and formal comparisons were conducted using appropriate statistical tests.

### 2.2. Study Design

Each nusinersen administration consisted of 4-month intervals according to the maintenance dose protocol of the CHERISH trial [23]. Baseline demographic data was collected and physiotherapy evaluations were performed before the initiation of nusinersen, and blood samples were collected from both groups to analyze serum SMN protein and IGF-1 levels.

After a month of baseline assessments, the training group underwent a 12-week aerobic exercise program using a bicycle ergometer, performed three times per week. In addition, both groups were prescribed the same home-based exercise program—including range-of-motion, stretching, and breathing exercises—which was recommended five days per week. Participants in the training group were supervised by a physiotherapist once per week in the clinic and received an additional weekly online session to ensure proper technique, safety, and adherence. The home-exercise group did not receive supervised aerobic training and was instead monitored through scheduled monthly clinical visits. Exercise adherence in both groups was assessed using structured exercise diaries, which were reviewed by physiotherapists during each clinical visit.

**Figure 1:**
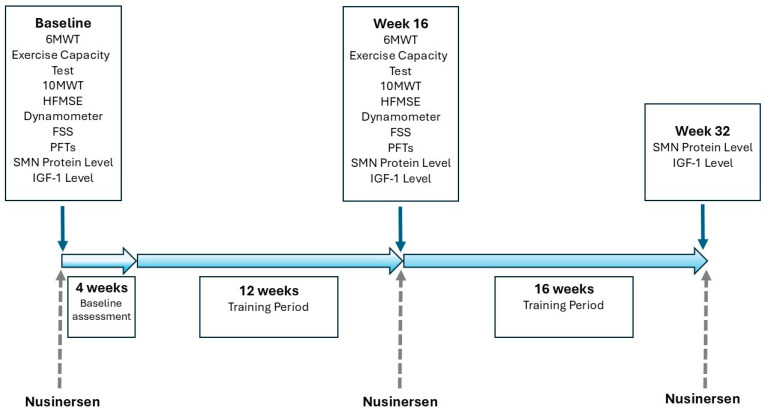
Study design is shown. 6MWT = six-minute walk test, 10MWT = 10-m walk test, FSS = Fatigue Severity Scale, HFMSE = Hammersmith Functional Motor Scale—Expanded, IGF-1 = insulin-like growth factor-1, PFTs = pulmonary function tests, SMN = survival motor neuron. Arrows indicate different aspects: thick blue arrows represent the main study timeline, thin blue arrows indicate assessment points, and dashed grey arrows indicate nusinersen applications.

After the 12-week training program, blood samples were collected again from both groups before the next nusinersen administration to measure SMN protein and IGF-1 levels, and clinical evaluations were repeated. After 12 weeks, the aerobic exercise training continued for another four months with the exercise frequency maintained at three sessions per week. At 8 months, blood samples were collected again to evaluate the long-term effects of exercise on SMN protein and IGF-1 levels (Figure 1).

To minimize potential confounding effects of nusinersen, blood samples were collected at standardized time points. Specifically, each sample was obtained in the morning of the scheduled nusinersen administration day, just before the injection. This ensured consistent timing at all three assessment points at the end of each 4-month treatment cycle for both groups.

### 2.3. Aerobic Exercise Training Protocol

Aerobic exercise training was conducted using a bicycle ergometer (Monark Model 839E, Monark, Vansbro, Sweden) at a moderate intensity of 60–70% of the maximum heart rate, monitored with a Polar chest strap. Training sessions were scheduled three days a week, 30 min per day, over 12 weeks. Each session included a 5-min warm-up, a gradual increase in workload until the target heart rate was reached, and a 5-min cooldown.

Patients used their own stationary bicycles at home. The target heart rate was calculated using the Karvonen formula [24]. Patients were taught to maintain a moderate intensity during sessions using the Borg Rating of Perceived Exertion scale (range 6–20), aiming for a perceived exertion level of 11–13. This scale also served as a complementary self-monitoring tool alongside heart rate tracking [25].

To avoid excessive fatigue, initial training sessions were conducted intermittently. Progression was achieved by increasing exercise duration: 1–4 weeks, 20 min total (10 min exercise + 5 min rest + 10 min exercise); 4–8 weeks, 30 min total (15 min exercise + 5 min rest + 15 min exercise); and weeks 8–12, 30 min continuous exercise.

To ensure safe and accurate implementation of the home-based exercise program, participants in the training group were encouraged to use their own smartwatches to monitor heart rate and were individually instructed during clinical visits on how to interpret the data.

Any adverse effects, complaints, or modifications in exercise intensity or duration were documented in exercise diaries and reviewed during clinical visits.

### 2.4. Outcome Measures

All patients were evaluated at baseline and post-training using objective outcome measures.

### 2.5. Primary Outcomes

Exercise Capacity Test: Exercise capacity was assessed using the ergometer (Monark Model 839E, Sweden) provided during training. A submaximal incremental exercise test, previously shown to be applicable in Post-Polio Syndrome (PPS), was performed [26]. Following a 2-min warm-up period without any load, the workload was increased by 5 to 10 W every minute. During the test, oxygen uptake (mL/kg/min) was recorded, and heart rate was monitored using a Polar chest strap. The test was terminated if heart rate exceeded 80% of the predicted maximum, if pedaling frequency dropped below 60 cycles per minute, or if the patient was unable to maintain pedaling speed.

Six-Minute Walk Test (6MWT): Conducted in accordance to ATS/ERS guidelines, patients were instructed to walk as quickly as possible for 6 min along a 30-m marked corridor, turning around cones and repeating the cycle [27,28]. A physiotherapist followed closely to ensure safety, allowing rest without sitting if fatigue occurred, and encouraging resumption of walking as soon as possible. The distance covered in meters was recorded at the end of the test.

### 2.6. Secondary Outcomes

Hammersmith Functional Motor Scale–Expanded (HFMSE): This scale evaluates motor function in SMA Type II and III patients, including 33 tasks assessing motor performance such as rolling, sitting, crawling, kneeling, standing, and walking. Each item is scored between 0 and 2. The maximum possible score in HFMSE is 66, with a higher score indicating a better functional status [29].

Pulmonary Function Test (PFT): Lung volumes were measured using a spirometer (MIR Spirobank G) following ATS/ERS guidelines [30]. Lung function was assessed by measuring forced vital capacity (FVC), forced expiratory volume in 1 s (FEV1), and peak expiratory flow (PEF) as a percentage of the predicted value and in liters, based on age and height. The best three attempts were recorded.

Hand-Held Dynamometry (HHD): Bilateral strength of the gastrocnemius and quadriceps muscles was measured using a dynamometer (MicroFET^®^, Hoggan Scientific, LLC, Salt Lake City, UT, USA). Patients were instructed to push the dynamometer with maximum effort while seated, holding the isometric contraction for 2–3 s. The best value from three trials for each side was recorded [31].

10-Meter Walk Test (10MWT): The time taken to walk 10 m as quickly and safely as possible was recorded using a stopwatch. Timing started when the front foot crossed the start line and stopped when the back foot crossed the finish line [32].

Fatigue Severity Scale (FSS): This is a nine-item patient-reported measure assessing the physical, social, and cognitive effects of fatigue over the past week, including the day of testing. Each item was scored between 0 and 7, with the total score averaged. Scores > 4 indicated abnormal fatigue, while scores > 5 indicated severe fatigue [33].

### 2.7. Analysis of IGF-1 in Serum Samples

The samples taken into blood collection tubes were centrifuged at 3000 rpm for 15 min. Aliquots of serum samples were stored at −80 °C until the time of analysis. Serum IGF-1 levels were measured by the enzyme-linked immunosorbent assay (ELISA) technique using an IGF-1 kit (Cat no: QS0940Hu/Sunlog Biotech Co., Ltd. Hangzhou, China) and analyzed according to the manufacturer’s instructions. Serum IGF-1 levels were expressed as ng/mL. The intra-assay and the interassay coefficients of variations (CV) of IGF-1 were 6.9% and 9.5%, respectively.

### 2.8. Western Blot Testing

Venous blood samples (2 mL) were collected from each participant and processed to isolate peripheral blood mononuclear cells (PBMCs), which were then stored at −80 °C. PBMCs samples were thawed and lysed in a buffer containing 20 mM Tris (pH 7.4), 137 mM NaCl, 2 mM EDTA (pH 7.4), 1% Triton X-100, 25 mM β-glycerophosphate, 1 mM phenylmethylsulfonyl fluoride (PMSF), and protease inhibitors. Lysates were centrifuged at 20,000× *g* for 20 min at 4 °C. Protein concentration was determined using a bicinchoninic acid (BCA) Protein Assay Kit (Pierce Biotechnology, Waltham, MA, USA). Equal amounts of protein (20 µg) were separated by 4–20% SDS-PAGE and transferred to a 0.45 μm polyvinylidene fluoride (PVDF) membrane at 100 V for 80 min. Membranes were blocked with 5% non-fat dry milk in PBST (10 mM sodium phosphate, 0.9% NaCl, and 0.1% Tween 20), followed by overnight incubation with primary antibodies against SMN1 (SMN1 monoclonal antibody, 2F1, MA5-15857 with predicted molecular weight of approximately 39 kDa/Invitrogen, Waltham, MA, USA) and GAPDH (predicted molecular weight of 37 kDa/Invitrogen, Waltham, MA, USA) in 1:500 dilutions. After washing, membranes were incubated for one hour with horseradish peroxidase (HRP)-conjugated secondary antibody at 1:5000 dilutions. Immunoreactivity of the protein bands was detected by enhanced chemiluminescent autoradiography (ECL kit, Invitrogen, Waltham, MA, USA). A molecular weight standard (Page Ruler Prestained Protein Ladder for 180^−10^ kDaA/Invitrogen, Waltham, MA, USA) was loaded to assess the relative molecular mass of detected bands. The immune blot bands were quantified by measuring band intensity with ImageJ software (Java-based image-processing and analysis software, version 1.54p, National Institutes of Health, Bethesda, MD, USA) using the same pixel scale for all pictures. Band intensities were normalized by GAPDH expression and expressed as arbitrary units.

### 2.9. Statistical Analysis

A priori power analysis was conducted using the PASS 2008 software to determine the required sample size. The calculation was based on oxygen uptake data reported by Orngreen et al. [34], who investigated the effects of a 12-week aerobic training program in patients with myotonic dystrophy. Assuming a mean difference of 4.8 units and a standard deviation of 2.77 units in oxygen uptake, the required sample size was calculated as six participants per group (80% power, 95% confidence) using a repeated-measures design with a compound symmetry covariance structure. In this study, both the six-minute walk distance and oxygen uptake were defined as co-primary outcome measures reflecting exercise capacity.

Descriptive statistics were given with medians (IQR) [interquartile range] and frequencies with percentages, as n (%). Since the sample sizes of the groups did not meet the assumptions of parametric tests, analyses were conducted using nonparametric methods. The Friedman test with the Dunn–Bonferonni post hoc test, the Mann–Whitney U test, the Pearson chi-square test, and linear mixed models using restricted maximum likelihood estimation were employed for between-group comparisons. Delta was calculated as delta (∆) = post-training − pre-training, and the Wilcoxon signed rank test was used for within-group comparisons. The data were analyzed using IBM SPSS Statistics (Version 30). Levels of significance were taken with *p* = 0.05.

## 3. Results

### 3.1. Demographic and Clinical Patient Characteristics

A total of 23 participants were included in this study, with 12 individuals (52.17%) in the training group and 11 individuals (47.83%) in the home-exercise group. The participants consisted of 11 males (47.83%) and 12 females (52.17%). The median age of the home-exercise group was 30.00 (21.00), while the median age of the training group was 28.50 (10.00).

Before aerobic training, the HFMSE score was 55.00 (13.00) in the home-exercise group and 58.50 (6.00) in the training group. Both groups were homogeneously distributed in terms of age, gender, height, weight, disease onset age (months), and motor function, showing no significant differences. There were also no statistically significant differences between the groups at baseline in key clinical outcome measures (e.g., 6-min walk distance, fatigue severity, pulmonary function parameters) (*p* > 0.05). The demographic, physical, and baseline clinical characteristics comparisons of training and home-exercise group are presented in Table 1.

In the training group, all 12 participants continued the program, and 9 of them (75.0%) completed the full 12-week intervention as prescribed. In the home-exercise (control) group, all 11 participants continued the program, and 9 of them (81.8%) fully adhered to the prescribed protocol. These adherence rates indicate high compliance across both groups and are included to ensure transparency regarding participant engagement.

In the training group, 10 patients reached the target session duration of 30 min after 12 weeks of training, while 2 patients achieved a session duration of 20 min. These two patients reached the target session duration of 30 min during the additional 4-month training period following the completion of the 12-week training program. During the 4-month training period, the session duration continued to be 30 min.

The baseline HHD-measured right gastrocnemius muscle strength (force in kg) was significantly higher in the home-exercise group compared to the training group (*p* = 0.044). Other demographic, physical, and pre-treatment clinical characteristics were similar between the groups (*p* > 0.05).

### 3.2. The Effect of 12 Weeks of Aerobic Exercise Training on Physical Function

In the exercise capacity test, both groups improved (*p* = 0.028 and *p* = 0.002), with a significantly greater increase in the training group compared to baseline (*p* < 0.001). The home-exercise group remained unchanged in the 6MWT, whereas the training group exhibited significant improvement (*p* = 0.003). Motor function assessed by HFMSE remained unchanged in both groups. In muscle strength measured by HHD, the home-exercise group remained unchanged, while the training group showed improvements in the strength of the right and left quadriceps (*p* = 0.005, *p* = 0.031) and the right gastrocnemius (*p* = 0.034). The change in the right quadriceps was found to be significantly higher in the training group (*p* = 0.004). In the 10MWT, the time required to complete the distance decreased (*p* = 0.019), and fatigue measured by FSS was also reduced (*p* = 0.005). There was a significant improvement in FSS in the training group (*p* = 0.037). Within-group and between-group comparisons are presented in Table 2.

In the pulmonary function test, percentage-predicted forced vital capacity (FVC) improved only in the training group (*p* = 0.016). Within-group and between-group comparisons are presented in Table 3.

### 3.3. The Effect of Aerobic Exercise Training on Blood SMN Protein and IGF-1 Levels

In the home-exercise group, SMN protein and IGF-1 levels remained unchanged across the pre-training, post-training first (12 weeks), and post-training second (28 weeks) time points (*p* = 0.651). In contrast, SMN protein levels in the training group varied significantly over time (*p* = 0.027), with a notable improvement at the second post-training measurement compared to baseline (*p* = 0.022). Supplementary Figure S1 illustrates that SMN protein expression was visibly increased in the training group following the training period, further supporting the ELISA-based findings (Supplementary Figure S1).

Similarly, IGF-1 levels in the training group showed significant changes across time points (*p* = 0.014), with a significant improvement from pre-training to the second post-training measurement (*p* = 0.016). Linear mixed model results estimated using restricted maximum likelihood (REML) are presented in Table 4.

## 4. Discussion

The benefits and risks of physical exercise for patients with motor neuron diseases have long been debated. This study investigated the effects of aerobic exercise training on clinical parameters and molecular responses of SMN protein and IGF-1 hormone in ambulatory SMA Type III patients. Due to the milder disease in SMA Type III, this group forms a suitable target population for strengthening and endurance exercise protocols [35].

Previous studies have reported that while aerobic exercise improves exercise performance in SMA patients, it does not significantly improve clinical measures of motor function, strength, and fatigue [16,17]. In our study, HFMSE scores remained unchanged in both groups after 12 weeks of training. In the training group, walking speed in the 10MWT improved from 1.37 m/s to 1.60 m/s following the intervention, corresponding to a 0.23 m/s increase (approximately 16.8%). Prior research has suggested that a change of ≥0.10 m/s represents a clinically meaningful improvement in populations with mobility limitations; thus, our findings exceed this threshold [36]. In the training group, a significant 31-m improvement was observed in the 6MWT. A ≥24-m change in walking distance has previously been identified as the minimal clinically important difference (MCID) for ambulatory individuals with SMA. Thus, the improvement in our cohort surpasses this threshold and reflects a meaningful enhancement in functional capacity [35].

Previous studies have not shown improvements in muscle strength measurements following aerobic exercise training. After 12 weeks of training, muscle strength in the right and left quadriceps and gastrocnemius remained stable in the home-exercise group. However, in the training group, following the 12-week aerobic exercise program, the strength of the right and left quadriceps increased by approximately 30%, while the strength of the right and left gastrocnemius improved by 13.8% and 15.0%, respectively. Previous literature suggests that increases greater than 10% in lower-extremity muscle strength are considered clinically meaningful. Therefore, the improvements observed in our study are not only statistically significant but also clinically and functionally relevant for ambulatory patients with SMA Type III [37]. In the exercise capacity, the change in the training group was 5.44 mL/kg/min. A clinically significant increase in oxygen uptake is considered to be 3.5 mL/kg/min [35]. The training group demonstrated a 34% improvement in exercise capacity, and the effect of training on oxygen uptake was clinically significant.

In the pulmonary function test, only a 4% improvement in FVC was observed in the training group after the exercise program. All tests and aerobic exercise sessions were well tolerated without any serious adverse events, and the protocol did not cause severe fatigue or pain during or after exercise sessions. Fatigue, measured by FSS, showed a clinically significant 24% improvement in the training group. The FSS score of 10 patients was below the abnormal fatigue threshold of <4.

Another aim of this study was to examine the molecular response of SMN protein and IGF-1 hormone to exercise in SMA Type III patients. The primary design of our study focused on a 12-week intervention period. However, the inclusion of a second post-training assessment aimed to observe how exercise-induced molecular adaptations change over time. SMN protein and IGF-1 levels remained unchanged after 12 weeks of training; however, the training group showed significant increases at the second follow-up compared to baseline.

A previous preclinical study demonstrated that exercise prolonged lifespan and protected motor neurons, effects associated with increased SMN expression in spinal cord tissue [12]. Similar tissue-specific molecular adaptations may have occurred in our study; however, since measurements were conducted using peripheral blood mononuclear cells (PBMCs), such changes may not have been detectable at week 12. Although PBMC analysis is a widely used and non-invasive method in clinical research, it may not fully reflect SMN expression in target tissues like motor neurons or the spinal cord.

Previous studies have indicated that peripheral SMN levels may serve as pharmacodynamic or prognostic biomarkers in SMA. The measurement of SMN mRNA and protein levels in peripheral blood has been shown to be technically reliable and correlated with SMN1/SMN2 gene copy number. Although these peripheral levels may not fully reflect central nervous system pathology, they offer accessible and practical biomarkers for monitoring biological responses [38].

IGF-1 is a growth factor that plays a fundamental role in muscle regeneration, motor neuron survival, and anabolic processes. Exercise has been shown to locally increase IGF-1 production, potentially supporting its neuroprotective effects on motor neurons. Elevated IGF-1 levels have been associated with prolonged motor neuron survival in neurodegenerative diseases [39]. In our study, the increase in IGF-1 levels observed at the second post-exercise measurement aligns with previous reports indicating that such biochemical changes typically occur following long-term and consistent exercise protocols [40]. These findings suggest that prolonged aerobic exercise may induce molecular adaptations linked to exercise-mediated physiological mechanisms.

There are various factors that can influence patients’ responses to exercise. The most important factor is selecting an exercise intervention appropriate to the patient’s baseline physical performance. Other important factors include characteristics such as age and gender. Our study includes a wide age range (18–57 years). The median age in the home-exercise group was 30, while in the training group, it was 29. The participants were primarily young adults. However, before the training, there were no differences between the groups in terms of baseline values for physical performance, age, and gender. Changes in IGF-1 and SMN protein expression should be evaluated considering the type, intensity, duration, and frequency of exercise. During the seven-month aerobic exercise training period, exercise frequency was three sessions per week. The progression strategy was limited in terms of gradually increasing exercise intensity and frequency to observe the long-term effects on physical function and molecular response. It has already been shown that excessive expression of IGF-1 leads to an increase in SMN protein levels in various tissues, including the spinal cord and muscles [41,42]. Future research may explore molecular pathways in more depth using methods such as muscle biopsy or cerebrospinal fluid (CSF) biomarkers. Although improvements were observed in our cohort regarding functional tests (6MWT, 10MWT) in the training group after the training period, HFMSE scores remained unchanged. Combining aerobic exercise with resistance strength exercises may yield better results in improving motor function.

Our study has some limitations. One of the main limitations is the lack of randomization, which may have introduced selection bias. To mitigate this risk, participants in the training and home-exercise groups were pair-matched based on age, sex, and baseline HFMSE score. While this matching approach improved group comparability at baseline, it may not fully eliminate residual confounding. Therefore, the results should be interpreted with this limitation in mind.

Baseline assessments showed no statistically significant differences between the two groups in terms of demographic, physical, and pre-treatment clinical characteristics, including age, sex, height, weight, and baseline functional status (*p* > 0.05), suggesting overall comparability. However, one exception was noted: the right gastrocnemius muscle strength measured by HHD was significantly higher in the home-exercise group (*p* = 0.044). This isolated difference may reflect the limitations of pair-matching in balancing all potential variables. Nevertheless, given the absence of differences in all other key variables, the impact of this single deviation on the study outcomes is likely minimal.

Another limitation is the difference in supervision intensity between groups. Participants in the aerobic training group were monitored more frequently through weekly in-person and online sessions, while the home-exercise group received only monthly clinical visits. Although both groups used structured exercise diaries to track adherence, the greater contact in the training group may have introduced performance bias. This potential source of bias should be considered when evaluating the findings.

Additionally, the home-based exercise protocol relied on self-monitoring using personal smartwatches and the Borg Rating of Perceived Exertion. While this combination allowed participants to regulate exercise intensity independently, it lacks the objectivity of supervised heart rate monitoring. The absence of real-time remote monitoring may have introduced variability in adherence and exercise execution, which could influence the outcomes.

Due to intracellular synthesis and the limited passage of SMN protein into the CSF, direct quantification in CSF is not feasible. As SMN protein levels in our study were measured in PBMCs, this may not directly reflect motor neuron expression within the central nervous system. However, ethical and practical constraints prevent direct tissue sampling in clinical settings. Nevertheless, the use of a control group enables the interpretation of significant systemic changes in SMN protein levels induced by aerobic exercise.

One of the limitations of the study is the limited participation of individuals with SMA Type III in long-term exercise intervention studies. This naturally affected the sample size. However, since the number of participants in our study was relatively small, larger, multi-center studies should be conducted.

Our findings showed that an aerobic exercise protocol may be useful and safe for SMA Type III patients and that 12-week moderate-intensity aerobic exercise training improves functional performance and strength, enhances exercise capacity, and reduces subjective fatigue. According to our results, this aerobic exercise protocol demonstrated its feasibility as an add-up treatment for SMA Type III patients.

## Figures and Tables

**Table 1:** Baseline comparison of demographic, physical, and clinical characteristics between the training and home-exercise groups.

	Training Group	Home-Exercise Group	*p*-Value
	(n = 12)	(n = 11)	
Age (year)	28.50 (10.00)	30.00 (21.00)	0.487
Height (cm)	169.50 (17.00)	167.00 (12.00)	0.928
Weight (kg)	62.50 (25.00)	56.00 (24.00)	0.449
BMI (kg/m^2^)	22.65 (4.30)	19.80 (6.30)	0.487
HFMSE Total	58.50 (6.00)	55.00 (13.00)	0.449
Age at symptom onset (month)	84.00 (123.00)	84.00 (96.00)	0.928
Gender			0.292
Male (n)	7 (58.30%)	4 (36.40%)	
Female (n)	5 (41.70%)	7 (63.60%)	
HFMSE Total	55.00 (12.00)	58.50 (6.00)	0.379
6MWT Distance (m)	307.00 (215.00)	382.50 (170.25)	0.413
Exercise Capacity (mL/kg/min)	9.62 (4.40)	14.65 (10.12)	0.374
FVC (% predicted)	86.00 (23.00)	90.00 (18.00)	0.740
FEV1 (% predicted)	92.00 (22.00)	95.50 (23.00)	0.976
PEF (% predicted)	87.00 (31.00)	81.00 (23.75)	0.449
FVC (L)	3.53 (1.42)	3.84 (1.79)	0.566
FEV1 (L)	3.08 (1.02)	3.51 (1.81)	0.566
PEF (L)	6.01 (3.79)	5.96 (2.21)	0.928
HHD Quadriceps right force (kg)	3.17 (2.00)	3.63 (1.87)	0.608
HHD Quadriceps left force (kg)	3.13 (2.04)	3.27 (1.98)	0.976
HHD Gastrocnemius right force (kg)	7.30 (1.86)	8.07 (1.19)	0.044
HHD Gastrocnemius left force (kg)	7.12 (1.32)	7.26 (1.21)	0.525
10MWT (s)	8.34 (4.20)	7.29 (1.85)	0.487
FSS	4.40 (1.20)	4.10 (1.48)	0.651
SNM Protein	0.70 (0.64)	0.65 (0.69)	0.674
IGF-1 (ng/mL)	3.40 (2.05)	4.09 (4.95)	0.190

The data were presented as median (IQR) [interquartile range]. BMI = body mass index. HMFSE = Hammersmith Functional Motor Scale—Expanded; n = sample size.

**Table 2:** Comparison of physical function outcome measures of the training and home-exercise groups after 12 weeks of aerobic exercise training.

		Training Group (n = 12)	Home-Exercise Groups (n = 11)	*p* Value
HFMSE Total	Pre-I	58.50 (5.75)	55.00 (12.00)	0.695 *
	Post-I	60.00 (6.75)	55.00 (13.00)	
	Δ	0.00 (1.50)	0.00 (0.00)	
	*p*	0.102 ^#^	0.180 ^#^	
6MWT Distance (m)	Pre-I	382.50 (170.25)	307.00 (215.00)	0.169 *
	Post-I	409.00 (186.00)	346.00 (230.00)	
	Δ	30.50 (16.75)	0.00 (49.00)	
	*p*	0.003 ^#^	0.213 ^#^	
Exercise Capacity (mL/kg/min)	Pre-I	14.65 (10.13)	9.60 (4.40)	<0.001 *
	Post-I	19.63 (12.59)	11.00 (6.60)	
	Δ	5.44 (4.48)	0.51 (1.47)	
	*p*	0.002 ^#^	0.028 ^#^	
HHD Quadriceps right force (kg)	Pre-I	3.63 (1.87)	3.17 (2.00)	0.004 *
	Post-I	4.78 (1.26)	3.10 (1.41)	
	Δ	1.00 (1.46)	0.04 (0.27)	
	*p*	0.005 ^#^	0.561 ^#^	
HHD Quadriceps left force (kg)	Pre-I	3.27 (1.98)	3.13 (2.04)	0.069 *
	Post-I	4.60 (2.08)	3.22 (1.77)	
	Δ	0.52 (1.73)	0.04 (0.54)	
	*p*	0.031 ^#^	0.859 ^#^	
HHD Gastrocnemius right force (kg)	Pre-I	8.07 (1.19)	7.30 (1.86)	0.104 *
	Post-I	9.18 (1.86)	7.93 (2.10)	
	Δ	0.95 (1.92)	0.00 (0.59)	
	*p*	0.034 ^#^	0.373 ^#^	
HHD Gastrocnemius left force (kg)	Pre-I	7.26 (1.21)	7.12 (1.32)	0.169 *
	Post-I	8.35 (1.29)	7.12 (1.35)	
	Δ	1.00 (2.29)	−0.18 (1.09)	
	*p*	0.068 ^#^	0.306 ^#^	
10MWT (seconds)	Pre-I	7.29 (1.85)	8.34 (4.20)	0.079 *
	Post-I	6.24 (3.21)	8.47 (3.30)	
	Δ	−1.15 (1.57)	−0.03 (0.97)	
	*p*	0.019 ^#^	0.722 ^#^	
FSS	Pre-I	4.10 (1.48)	4.40 (1.20)	0.037 *
	Post-I	3.20 (1.05)	4.20 (0.50)	
	Δ	−0.90 (1.25)	−0.20 (0.90)	
	*p*	0.005 ^#^	0.476 ^#^	

The data were presented as median (IQR) [interquartile range]. *p* * = comparisons with delta (Δ) = Post-I–Pre-I. *p* ^#^: comparisons with pre-post within group. 6MWT = Six-minute walk test, 10MWT = 10-m walk test, FSS = Fatigue Severity Scale, HFMSE = Hammersmith Functional Motor Scale—Expanded, HHD = hand-held dynamometry, kg = kilogram, m = meter, min = minute.

**Table 3:** Comparison of PFT parameters of the training and home-exercise groups after 12 weeks of aerobic exercise training.

		Training Group (n = 12)	Home-Exercise Groups (n = 11)	*p* Value
FVC (% predicted)	Pre-I	90.00 (18.00)	86.00 (23.00)	0.091 *
	Post-I	92.50 (17.25)	88.00 (23.00)	
	Δ	3.50 (4.00)	−2.00 (8.00)	
	*p* value	0.016 ^#^	0.475 ^#^	
FEV1 (% predicted)	Pre-I	95.50 (23.00)	92.00 (22.00)	0.190 *
	Post-I	95.00 (21.00)	90.00 (22.00)	
	Δ	0.50 (2.75)	−4.00 (8.00)	
	*p* value	0.535 ^#^	0.247 ^#^	
PEF (% predicted)	Pre-I	81.00 (23.75)	87.00 (31.00)	0.880 *
	Post-I	74.50 (26.75)	84.00 (16.00)	
	Δ	−2.00 (12.25)	0.00 (19.00)	
	*p* value	0.783 ^#^	0.838 ^#^	
FVC (L)	Pre-I	3.84 (1.79)	3.50 (1.40)	0.169 *
	Post-I	4.02 (2.03)	3.70 (0.70)	
	Δ	0.09 (0.18)	−0.11 (0.38)	
	*p* value	0.116 ^#^	0.398 ^#^	
FEV1 (L)	Pre-I	3.51 (1.81)	3.08 (1.02)	0.169 *
	Post-I	3.58 (1.60)	3.06 (1.04)	
	Δ	0.02 (0.35)	−0.10 (0.26)	
	*p* value	0.505 ^#^	0.213 ^#^	
PEF (L)	Pre-I	5.96 (2.21)	6.01 (3.79)	0.833 *
	Post-I	6.56 (2.54)	6.01 (1.74)	
	Δ	−0.10 (1.06)	−0.06 (1.40)	
	*p* value	0.814 ^#^	1.000 ^#^	

The data were presented as median (IQR) [interquartile range]. *p* *: comparisons with delta (Δ) = Post-I–Pre-I. *p* ^#^: comparisons with pre-post within group. FEV1 = forced expiratory volume in 1 s, FVC = forced vital capacity, PEF = peak expiratory flow, PFT = pulmonary function test, L = liter.

**Table 4:** Comparison of SMN protein and IGF-1 values of the training and home-exercise groups after aerobic exercise training in the 1st and 2nd measurements.

		Training Group (n = 12)	Home-Exercise Groups (n = 11)	*p* Value *
SMN Protein	Pre-I	0.65 (0.70)	0.70 (0.64)	0.298 ^a^
	Post-I	1.18 (1.07)	0.94 (0.91)	
	Post-II.	1.42 (2.20)	1.11 (0.83)	
*p* value		0.027 ^c^	0.651 ^c^	
	Pre-I.–Post-I	0.539 ^b^		
	Pre-I–Post-II.	0.022 ^b^		
	Post-I–Post-II	0.539 ^b^		
IGF-1 (ng/mL)	Pre-I	4.09 (4.95)	3.40 (2.05)	0.900 ^a^
	Post-I	4.04 (3.98)	3.69 (1.21)	
	Post-II	8.41 (14.24)	4.12 (1.57)	
*p* value		0.014 ^c^	0.169 ^c^	
	Pre-I–Post-I	1.000 ^b^		
	Pre-I–Post-II	0.016 ^b^		
	Post-I–Post-II	0.172 ^b^		

The data were presented as median (IQR) [interquartile range]. *p* *: between-group comparisons based on delta (Δ = Post-I − Pre-I) values. ^a^: *p*-values obtained from linear mixed models using restricted maximum likelihood estimation. SMN = survival motor neuron, IGF-1 = insulin-like growth factor- 1. ^b^: *p*-values obtained from Dunn–Bonferonni post hoc test. ^c^: *p*-values obtained from Friedman test.

## Data Availability

The datasets generated during and/or analyzed during the current study are available from the corresponding author on reasonable request.

## Supplementary Materials

The following supporting information can be downloaded at https://www.mdpi.com/article/10.3390/jcm14176087/s1.
