# Supplementary material for: The Effect of Aerobic Exercise Training on Patients with Type III Spinal Muscular Atrophy
Source: J Clin Med. 2025 Aug 28;14(17):6087. doi: 10.3390/jcm14176087 (PMC12429496; doi:10.3390/jcm14176087)
Supplement: Supplementary file 1 [file jcm-14-06087-s001.zip › jcm-3733683-supplementary.pdf]

Supplementary Materials.

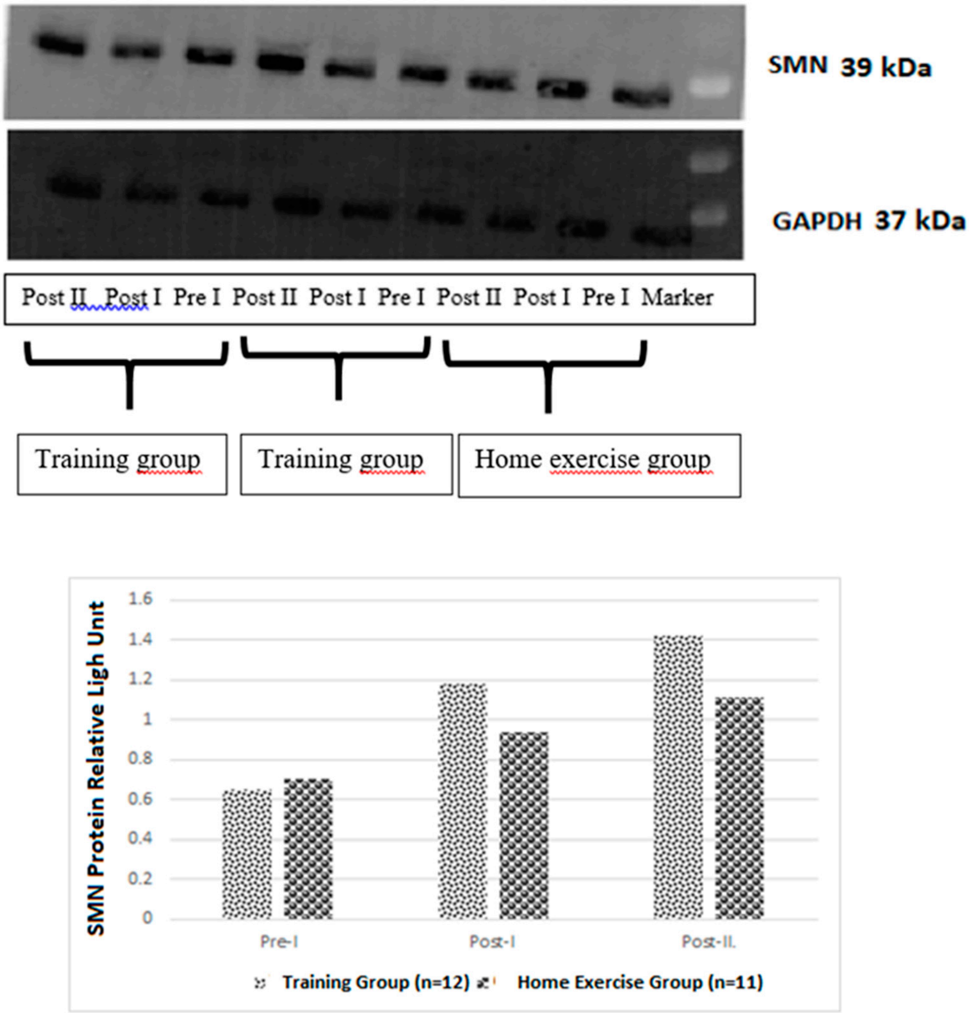

Figure S1. Examples from training and home exercise group patients from the SMN protein band density analyses in the training groups at the pre I (Initial), Post-I. (12th week after training), and Post-II (7 month after training) measurements after aerobic exercise (1A). Column chart showing the evaluation of data from all participants and the relationship between groups (1B). The SMN protein level increased at the post-training second measurement (Post II) compared to the pre-training level (Pre I) (p=0.022).Data are presented as median (IQR) [interquartile range].
